# ncRNA-mediated upregulation of *FAM83A* is associated with poor prognosis and immune infiltration in pancreatic cancer

**DOI:** 10.3389/fendo.2023.1093042

**Published:** 2023-03-31

**Authors:** Wenbo Zou, Hao Wang, Dingguo Wu, Yunyang Wu, Kuiping Zhou, Yuanshu Lian, Gengyun Chang, Yuze Feng, Jifeng Liang, Gao Huang

**Affiliations:** ^1^ Department of General Surgery, No.924 Hospital of PLA Joint Logistic Support Force, Guilin, China; ^2^ Neurology Department, No.924 Hospital of PLA Joint Logistic Support Force, Guilin, China

**Keywords:** *FAM83A*, pancreatic cancer, prognosis, immune cell infiltration, immunotherapy

## Abstract

**Introduction:**

Malignant pancreatic cancer has poor long-term survival. Increasing evidence shows that *FAM83A* (family with sequence similarity 83 member A) plays a vital role in tumorigenesis and malignant progression in some human cancer types. The present study explored the potential mechanism of *FAM83A* in improving the prognosis of pancreatic cancer patients.

**Methods:**

Transcriptomic and clinical data from patients were obtained from The Cancer Genome Atlas while *FAM83A* expression was measured in tumorous pancreatic tissue compared with normal controls by quantitative real-time PCR and immunohistochemistry.

**Results:**

*FAM83A* is a vital prognostic indicator and potential oncogene in pancreatic cancer via pan-cancer analysis. *In silico* analysis revealed that AL049555.1/hsa-miR-129-5p axis was the pivotal upstream ncRNA- mediated pathway of *FAM83A* in pancreatic cancer. Furthermore, *FAM83A* expression was related to immune cell infiltration through vital immune-related genes including *programmed cell death 1 (PDCD1)*, and tumorigenesis through common mutation genes including *KRAS protooncogene GTPase (KRAS)*, and *SMAD family member 4 (SMAD4)*. In summary, ncRNA-mediated upregulation of *FAM83A* is associated with poor long-term survival and immune cell infiltration in pancreatic cancer.

**Discussion:**

*FAM83A* may be used as a novel survival-related and immune-related biomarker. This information suggests that *FAM83A* may be a novel therapeutic target for combined or individual treatment for patients with pancreatic cancer.

## Introduction

Pancreatic cancer (PC) has the highest mortality rates of all the cancer types, with an average 5-year survival rate of approximately 10% in the United States (1). A lack of obvious early symptoms and a difficult diagnosis led to poor treatment efficacy and prognosis, thus increasing mortality. Radical surgery is the only curative treatment, and adjuvant chemotherapy is currently the main treatment for advanced PC. The increasing development of various comprehensive treatments has gradually increased the overall survival (OS) of patients (1, 2); however, most patients lose the opportunity for surgical treatment because of distant metastasis or local invasion, and resistance to chemotherapy is inevitable (3, 4). Immunotherapy has recently revolutionized oncotherapy since it can mobilize the patient’s immune system to enhance its antitumor abilities (5). New human cancer-immune phenotypes were proposed: immune-inflamed, immune-excluded, and immune-desert tumors (6) highlighting that immune subtypes affect anticancer responses. Thus, exploring the biomarkers that participate in immune-subtype transition is essential to better understand the immunologic mechanisms and assist the immunotherapy of patients with PC. Our previous studies showed that several key genes play a vital role in immune subtype transition *via* plentiful bioinformatic analyses (7). According to the previous study, we explored the differential expressed markers among subtypes, and found the *FAM83A* play an essential role in the tumorigenesis and immune mechanism of pancreatic cancer. It may exert an effectiveness on the immune subtype transition. However, its specific prognostic value and impact of immune microenvironment in various cancers involved PC need to be further revealed and validated.

Family with sequence similarity 83 member A (*FAM83A*) is located on chromosome 8q24 and is a potential biomarker in human cancer (8). The conserved DUF1669 domain at the N-terminus participates in tumorigenesis and malignant progression of several human cancers (9–11). *FAM83A* is upregulated in various malignancies such as lung, breast, and cervical cancers (12–15) indicating that it is a biomarker of poor prognosis and suggests that *FAM83A* is a key oncogene (16). *FAM83A* depletion play an important role in breast cancer, *FAM83A* can reverse the malignant biological behaviors, and rendered the sensitive to EGFR-tyrosine kinase inhibitors (17). *FAM83A* upregulation promotes the progression of non-small cell lung cancer (NSCLC) by inhibiting the mitogen-activated protein kinase (MAPK) signal transduction pathway (12), and promotes tumorigenicity in NSCLC *via* the signal-regulated kinase (ERK) pathway and phosphatidylinositol-3-kinase/mammalian target of rapamycin (PI3K/Akt/mTOR) pathways (18). In addition, FAM83A/PI3K/AKT/c-Jun formed a feedback structure that induced malignant progression in hepatocellular carcinoma (19). *FAM83A* was also studied in PC: Upregulated *FAM83A* promotes cancer stem cell-like traits and enhances chemoresistance in PC showing that *FAM83A* plays a vital oncogenic role in the malignant progression of PC (20). Notably, *FAM83A* upregulates *tetraspanin 1* (*TSPAN1*) through the WNT-CTNNB1 signaling pathway and affects macroautophagy/autophagy, suggesting that *FAM83A* and its target axis may predict prognosis and become a novel therapeutic target for patients with PC (21). Although several studies have revealed the mechanism of action of *FAM83A* in PC, but lack of some researches explore the upstream regulators of *FAM83A* in PC. Thus, more research is needed to explore the possible regulated pathways of *FAM83A* for assisting treatment in patients with this disease.

In the present study, the prognostic value of *FAM83A* in pan-cancer was also revealed using expression and survival analyses. The candidate noncoding RNAs (ncRNAs); microRNAs (miRNAs) and long noncoding RNAs (lncRNAs) regulating *FAM83A* were detected in PC. Clinical correlation analyses of *FAM83A* expression identified the relationship of *FAM83A* expression with clinicopathologic characteristics and immune cell infiltration in PC. Finally, the correlation between *FAM83A* expression and key immune-related biomarkers and common mutation genes was investigated. In conclusion, ncRNA-mediated upregulation of *FAM83A* was associated with poor prognosis and immune status transition in PC patients.

## Materials and methods

### Data collection and preprocessing

The mRNA matrixes of 33 cancer types were downloaded from The Cancer Genome Atlas (TCGA) database (https://genome-cancer.ucsc.edu/) (22) and normalized for subsequent analyses. *FAM83A* differential expression analysis was performed in pan-cancer using “limma” package (23) with the threshold P value set to < 0.05. The GEPIA database (http://gepia.cancer-pku.cn/) was used to verify the differential expression of *FAM83A* in human cancer types (24).

Tumor tissues with pathological diagnosis of PC and paracancerous normal tissues were prospectively collected from the No.924 Hospital of PLA Joint Logistic Support Force, some of them were preserved in 10% neutral formalin-fixed for immunohistochemical (IHC) staining, and the other parts were preserved at -80°C refrigerator for quantitative real time-polymerase chain reaction (qRT-PCR). This study was approved by the ethics committee of the No.924 Hospital of PLA Joint Logistic Support Force. The patients/participants provided their written informed consent to participate in this study.

### Survival and clinical correlation analysis in pan-cancer

The predictive power of *FAM83A* in OS was produced using a Kaplan-Meier survival curve with “survival” and “survminer” R packages. Univariate Cox regression analyses evaluated the significance of *FAM83A* in predicting OS, disease-specific survival (DSS), disease-free interval (DFI), and progression-free interval (PFI) in screened cancer types. In addition, the correlation between *FAM83A* expression and the American Joint Committee on Cancer (AJCC) stage was analyzed in PC.

### Quantitative real-time PCR

Tumor tissues with pathological diagnosis of PC and normal tissues were prospective collected from the No.924 Hospital of PLA Joint Logistic Support Force. TRIzol reagent(Ambion) was used to extracted total RNA; NanoPhotometer^®^ C40 Touch (IMPLEN) was used to assess the RNA purity based on the ratio of OD260/280 and 260/230; Eppendorf Mastercycler^®^ was used to perform reverse transcription of qualified RNA to single-stranded complementary DNA according to the manufacturer’s instructions; StepOnePlus Real-Time PCR system was used to implement real-time quantification; *18S rDNA* was used as an internal reference, the cycle threshold (Ct) was recorded, and relative expression was calculated using the 2^−ΔΔCt^ method. *FAM83A, AL049555.1, hsa-miR-129-5p and 18S rDNA* primer sequences are shown in **Table S1**.

### Immunohistochemistry staining

Next, the PC, paracancerous and normal tissues preserved in in 10% neutral formalin-fixed were undergone IHC. The IHC staining of the collected tissue was performed according to the manufacturer’s protocol; the primary antibody is stated in **Table S2**. All images were obtained with an Olympus DP72 camera using Olympus image analysis software.

### Independent prognostic value of *FAM83A*

Univariate and multivariate Cox regression analyses were performed on the *FAM83A* expression level and clinical data including age, sex, race, and AJCC stage: T stage, N stage, and M stage. A nomogram was developed by combining the independent prognostic factors to predict the 1-, 2-, and 3-year survival of patients with PC, with calibration curves and receiver operating characteristic (ROC) curves generated to evaluate the performance of the nomogram. Analyses were performed using the “rms” and “foreign” R packages.

### Candidate miRNA prediction

Candidate upstream binding miRNAs of *FAM83A* were predicted based on the seven prediction programs: PITA, RNA22, miRmap, microT, miRanda, PicTar, and TargetScan in starBase. miRNAs predicted by two or more programs were included in subsequent analyses. Expression correlation analysis of target miRNAs with *FAM83A* in PC was conducted using starBase (http://starbase.sysu.edu.cn/) (25) according to the competing endogenous RNA (ceRNA) hypothesis (26). Final regulatory networks were elucidated using Cytoscape version 3.7.2. Potential binding of lncRNAs to the pivotal miRNAs was also predicted by starBase.

### Differential expression and survival analysis of predicted lncRNAs

GEPIA was used to evaluate the differential expression and prognostic value of candidate lncRNAs in PC by integrating transcriptomes from cancer patients and normal patients from the TCGA and Genotype-Tissue Expression (GTEx) portals (24).

### Immune cell infiltration analysis using CIBERSORT and tumor immune estimation resource database

*FAM83A* may participate in immune-subtype variation since it is significantly differentially expressed among the immune subtypes (7). The CIBERSORT algorithm was utilized to assess ICI in PC and estimate its distribution in 22 immune cell types by gene expression analysis (27). Spearman’s correlation analysis was used to evaluate the degree of correlation between *FAM83A* expression and ICI.

The correlation of *FAM83A* copy number variation with immune cell infiltration in PC was analyzed by TIMER (https://cistrome.shinyapps.io/timer/) (28) using a threshold of P < 0.05.

### Analysis of immune-related biomarkers and gene mutation correlation

Immune-related genes including immunostimulatory factors, immunoinhibitory factors, chemokines, and chemokine receptors were extracted from the Tumor and Immune System Interaction Database (TISIDB) (http://cis.hku.hk/TISIDB/) and used to determine correlation coefficients using R software (29). In addition, common gene mutations were also used in the analysis.

### Statistical analysis

R software (version 4.0.2), GraphPad Prism (version 8.0.1), and relevant website tools were used for all statistical analyses and graphics. Survival curves were generated using the Kaplan-Meier method and compared using the log-rank test. Statistical significance was set at P < 0.05 and all tests were two-tailed.

## Results

### *FAM83A* differential expression analysis in pan-cancer

**Figure 1:**
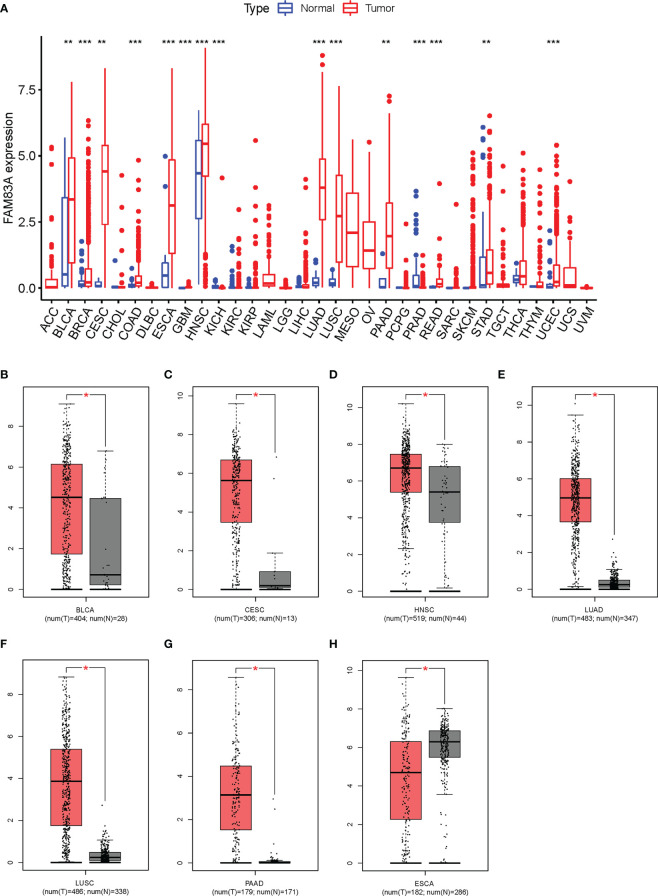
Expression analysis for FAM83A in multiple cancers: **(A)** The expression of FAM83A in human cancers based on TCGA database. **(B–H)** The differential expression of FAM83A between tumor and normal tissues in TCGA and GTEx: **(B)** BLCA **(C)**, CESC **(D)**, HNSC **(E)**, LUAD **(F)**, LUSC **(G)**, PAAD **(H)**, ESCA. *p value < 0.05; **p value < 0.01; ***p value < 0.001.

*FAM83A* was significantly differentially expressed in 15/33 types of human cancer analyzed from the TGCA database. Significantly high expression was observed in BLCA (bladder urothelial cancer), BRCA (breast invasive cancer), CESC (cervical squamous cell carcinoma and endocervical adenocarcinoma), COAD (colon adenocarcinoma), ESCA (esophageal carcinoma), GBM (glioblastoma multiforme), HNSC (head and neck squamous cell carcinoma), LUAD (lung adenocarcinoma), LUSC (lung squamous cell carcinoma), PAAD (pancreatic adenocarcinoma), READ (rectum adenocarcinoma), STAD (stomach adenocarcinoma), and UCEC (uterine corpus endometrial carcinoma) cancer tissues, while significantly low expression was found in PRAD (prostate adenocarcinoma) and KICH (kidney chromophobe) compared with the normal tissue (**Figure 1A**).

*FAM83A* expression levels in these 15 cancer types were reanalyzed using the GEPIA database with significantly higher expression observed in BLCA, CESC, HNSC, LUAD, LUSC, and PAAD cancer tissues and low expression in ESCA (**Figures 1B–H**). No significantly differentially expressed in other 8/15 types of human cancers (**Figure S1**). ESCA was omitted from further analysis since *FAM83A* expression was inconsistent between these two methods. These results suggested that upregulated *FAM83A* may play a crucial role in tumorigenesis.

### *FAM83A* prognostic value in screened cancers

**Figure 2:**
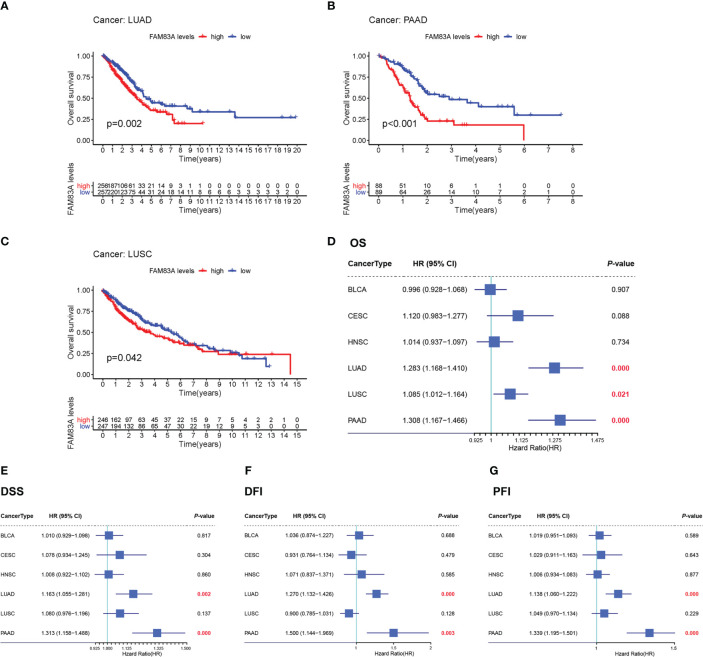
The overall survival (OS) and univariate Cox regression analysis for FAM83A in 6 human cancers based on TCGA: **(A–C)** The OS plot of FAM83A in LUAD **(A)**, PAAD **(B)**, LUSC **(C)**. **(D)** the OS forest plot. **(E)** the DSS forest plot. **(F)** the DFI forest plot. **(G)** the PFI forest plot.

Kaplan-Meier OS survival analysis for *FAM83A* in BLCA, CESC, HNSC, LUAD, LUSC, and PAAD showed that *FAM83A* was a positive risk factor for LUAD, LUSC, and PAAD patients (**Figures 2A–C**). Univariate Cox regression OS analyses suggested that *FAM83A* is a risk factor for patients with LUAD, LUSC, and PAAD in these six human cancer types (**Figure 2D**), while DSS, DFI, and PFI analyses revealed that *FAM83A* was a risk factor for patients with LUAD and PAAD (**Figures 2E–G**).

### qRT-PCR and immunohistochemistry

**Figure 3:**
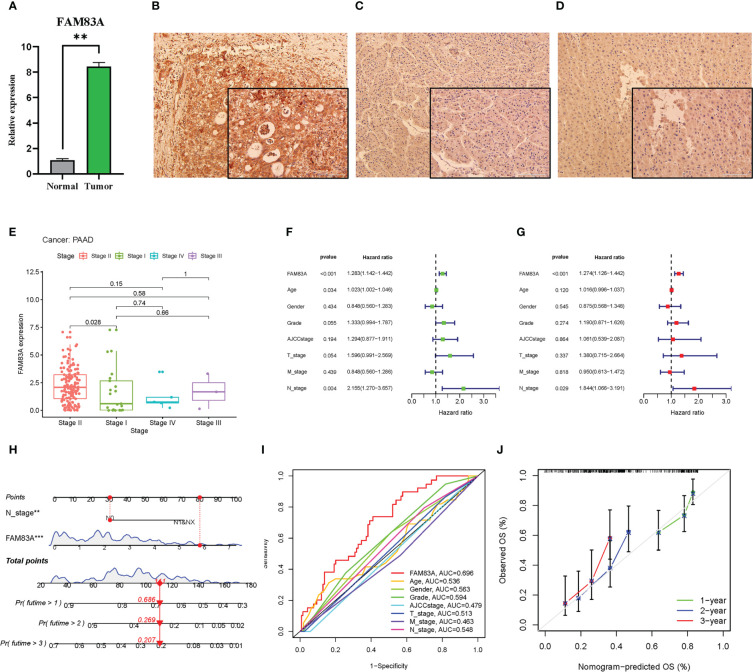
The laboratory experiment and clinical significance of FAM83A in PAAD: **(A–D)** The laboratory experiment. **(A)** The qRT-PCR result of FAM83A between the tumor and normal tissues. **(B)** The Immunohistochemistry (IHC) staining plot in PC. **(C)** The IHC staining plot in para-cancerous tissue. **(D)** The IHC staining plot in normal tissue. **(E-J)** clinical significance of FAM83A. **(E)** The correlation analysis of FAM83A with AJCC stage. **(F)** Univariate Cox regression analysis. **(G)** Multivariate Cox regression analysis. **(H)** The nomogram based on FAM83A expression and N stage. **(I)** The multivariate ROC curves. **(J)** The calibration curves at 1-, 2-, 3- years. **p value < 0.01; ***p value < 0.001.

*FAM83A* was significantly highly expressed in tumorous PC tissues obtained from patients compared with that in normal controls (**Figure 3A**). The IHC results showed that the *FAM83A* located in cytoplasm, while *FAM83A* was also positively expressed in PC compared with paracancerous and normal tissues (**Figures 3B–D**).

### Independent prognosis analysis

Increased *FAM83A* expression was found in late-stage PAAD after clinical correlation analysis of different AJCC stages in these six cancer types (**Figure 3E**). This indicated that *FAM83A* may be considered a detrimental prognostic biomarker in PC patients. Incorporation of clinicopathologic characteristics into the prognosis analysis showed that *FAM83A* and the N stage significantly correlated with OS in both univariate and multivariate Cox regression analyses (**Figures 3F, G**: P < 0.001 and P = 0.029, respectively). Integration of *FAM83A* and N stages into a risk model for predicting 1-, 2-, and 3-year survival rates, and *FAM83A* was the strongest prognostic indicator compared with other variates with an 0.696 area under curve (AUC) value of multivariate ROC (**Figures 3H, I**). The calibration curves confirmed that the nomogram adequately predicted the OS probability at 1-, 2-, and 3- years (**Figure 3J**) indicating that *FAM83A* is a good predictive indicator for patients with PC.

### Prediction of *FAM83A* upstream miRNAs

**Figure 4:**
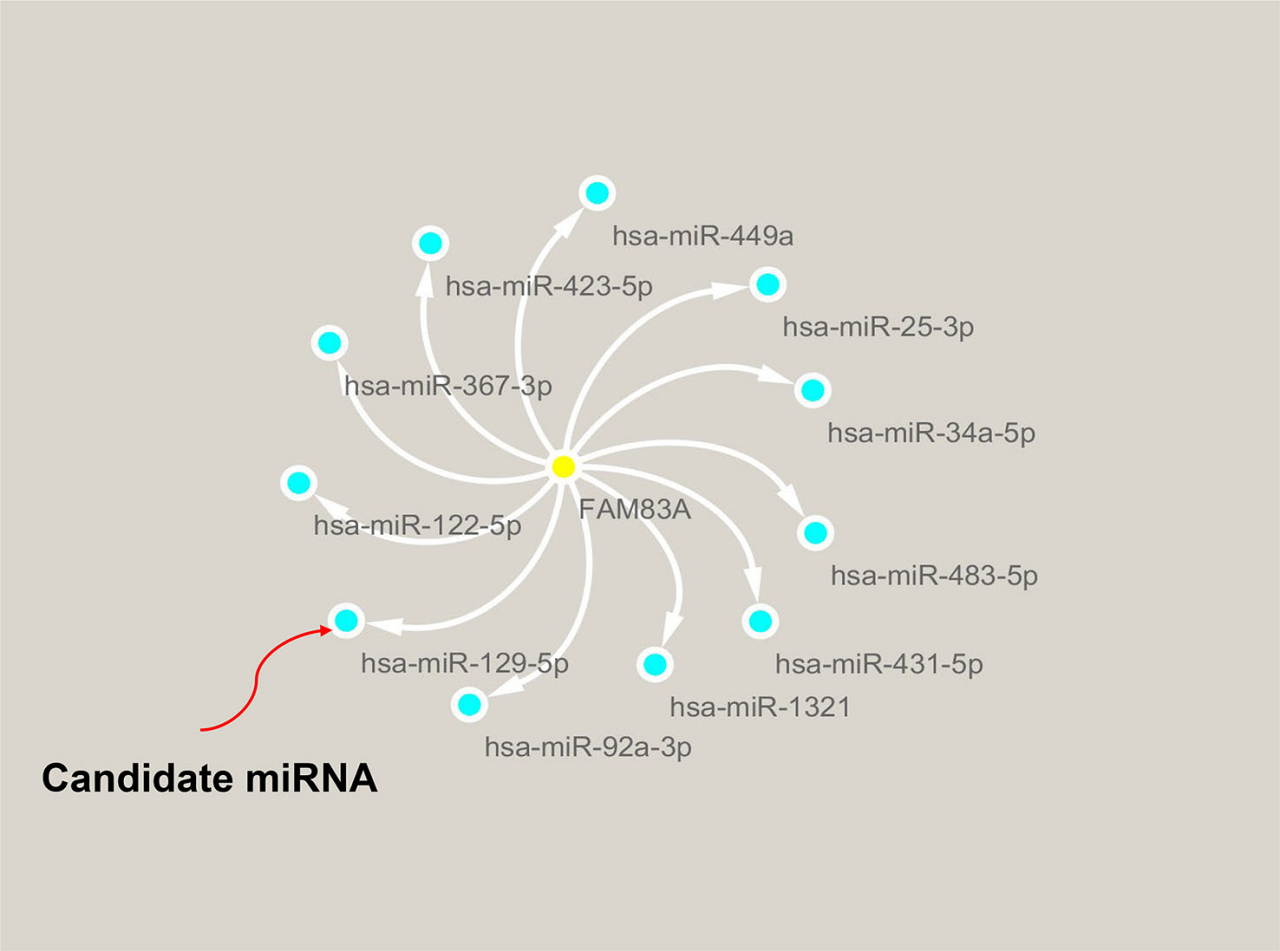
Identification of hsa−miR−129−5p as a potential upstream miRNA of FAM83A in PC.

ncRNA-related pathways play pivotal roles in gene expression with miRNAs inhibiting target gene expression (30). It was predicted that eleven upstream miRNAs bind to *FAM83A* (**Table S3**), and Cytoscape established the miRNA-FAM83A regulatory network (**Figure 4**). *FAM83A* significantly negatively correlated with *hsa-miR-129-5p* in PC by expression correlation analysis (**Table 1**, r = -0.322, P < 0.001). There was a statistically significant correlation between *hsa-miR-367-3p* and *FAM83A*, however, the correlation coefficient was weak.

**Table 1:** Correlation analyses between candidate miRNA and FAM83A.

miRNA name	Gene name	cor	P value	significance
hsa-miR-25-3p	FAM83A	0.104	1.66E-01	
hsa-miR-92a-3p	FAM83A	0.11	1.43E-01	
hsa-miR-129-5p	FAM83A	-0.322	1.19E-05	***
hsa-miR-34a-5p	FAM83A	0.067	3.74E-01	
hsa-miR-122-5p	FAM83A	-0.075	3.22E-01	
hsa-miR-367-3p	FAM83A	-0.171	2.23E-02	*
hsa-miR-449a	FAM83A	0.086	2.54E-01	
hsa-miR-431-5p	FAM83A	-0.019	8.00E-01	
hsa-miR-423-5p	FAM83A	-0.057	4.50E-01	
hsa-miR-483-5p	FAM83A	0.143	5.61E-01	
hsa-miR-1321	FAM83A	0	1.00E+00	

*p value < 0.05; ***p value < 0.001.

### Prediction of *hsa-miR-129-5p* upstream lncRNAs

**Figure 5:**
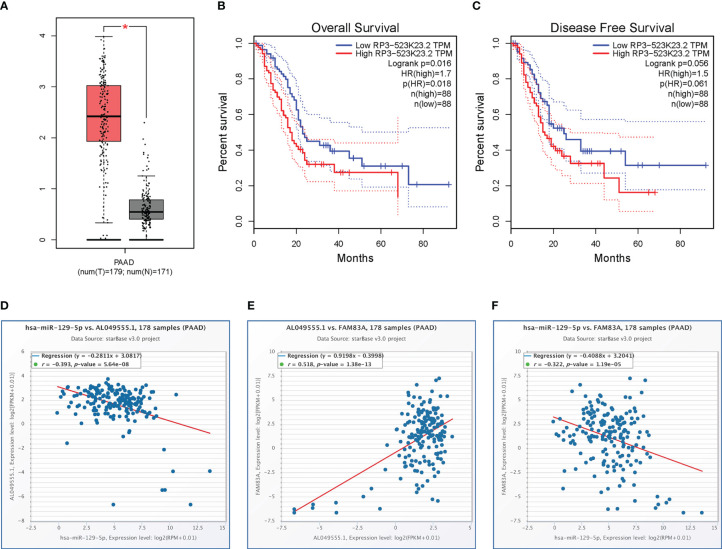
Expression analysis and survival analysis for AL049555.1, and relationship among AL049555.1/hsa−miR−129−5p/FAM83A axis: **(A)** The expression of AL049555.1 based on TCGA and GTEx. **(B-C)** survival analysis of OS and RFS for AL049555.1. **(D)** The AL049555.1 was strong negatively corelated with hsa-miR-129-5p. **(E)** The AL049555.1 was strong positively corelated with FAM83A. **(F)** hsa-miR-129-5p was strong negatively corelated with FAM83A. *p value < 0.05.

The starBase database identified a total of 118 potential upstream lncRNAs of *hsa-miR-129-5p* (**Table S4**) and a lncRNA-hsa-miR-129-5p regulatory network was established using Cytoscape (**Figure S2**). Differential expression and prognostic values of these lncRNAs in PC analyzed by the GEPIA database showed that only *AL049555.1* (RP3-523K23.2, ENSG00000261116) was significantly upregulated in PC compared with normal controls (**Figure 5A**). Increased *AL049555.1* expression indicated poorer OS and relapse-free survival in PC, even though P > 0.05 in the latter (**Figures 5B–C**). *AL049555.1* was strongly negatively correlated with hsa-miR-129-5p (r = -0.393, P value < 0.001) and strongly positively correlated with *FAM83A* (r = 0.518, P value < 0.001) (**Figures 5D, E**). Meanwhile, *hsa-miR-129-5p* was strongly negatively correlated with *FAM83A* (r = -0.322, P value < 0.001) (**Figure 5F**). This survival and correlation analysis suggested that the novel lncRNA, *AL049555.1*, may be the upstream lncRNA of the hsa-miR-129-5p/FAM83A axis in PC. Finally, in the qRT-PCR assay, *hsa-miR-129-5p* was significantly lowly expressed in tumorous PC tissues compared to that in normal controls, and *AL049555.1* was significantly highly expressed in tumorous PC tissues obtained from patients compared with that in normal controls (**Figure S3**), which shows the ncRNAs (*hsa-miR-129-5p* and *AL049555.1*) play the pivotal roles in tumorigenesis and progression of PC, and may be considered to have abilities to regulate the expression of *FAM83A*.

### ICI analysis of *FAM83A* in PC

**Figure 6:**
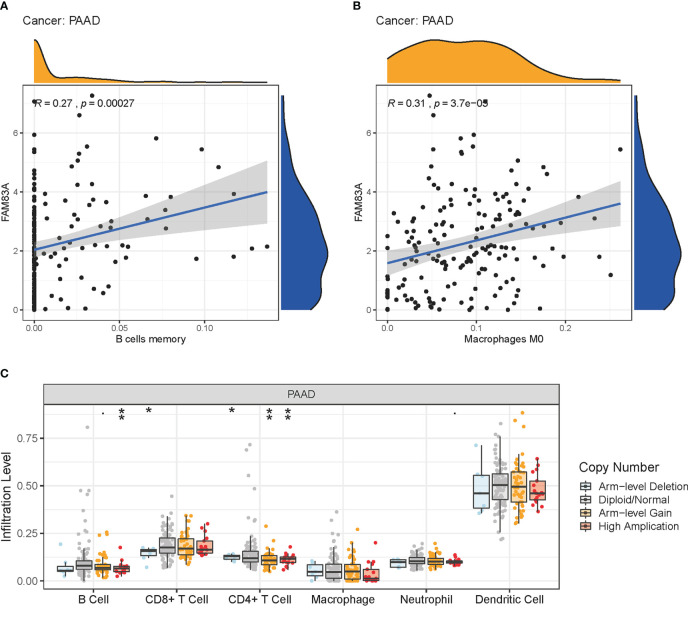
Immune cell infiltration (ICI) analysis: **(A-B)** The relationship of FAM83A expression with ICI in PC. **(A)** Memory B cells. **(B)** Macrophages M0. **(C)** ICI level under copy numbers variation of FAM83A in PC. *p value < 0.05; **p value < 0.01.

*FAM83A* expression was significantly positively correlated with memory B cells and M0 macrophage infiltration in PC, with significant changes in B cells, CD4+ T cells, and CD8+ T cell infiltration levels under various *FAM83A* copy numbers in PC (**Figures 6A–C**). This suggested that *FAM83A* variation may influence ICI in the tumor microenvironment of PC.

### *FAM83A* expression correlation with immune-related biomarkers and mutation-related genes in PC

**Figure 7:**
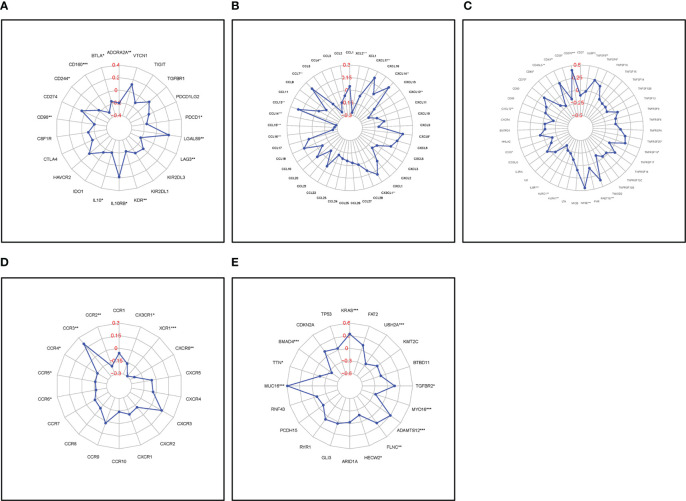
Correlation of FAM83A expression with immune-related biomarkers and common mutation genes: **(A)** Immunostimulatory factors. **(B)** Immunoinhibitory factors. **(C)** Chemokines. **(D)** Chemokine receptors. **(E)** Mutation genes. Red numbers are considered correlation coefficients.

*FAM83A* expression correlated with a series of immune-related genes in PC such as *CD160* and *interleukin 10* (*IL10*), and a vital immune checkpoint protein, *programmed cell death 1* (*PDCD1*); Chemokines, *C-C motif chemokine ligand 4* (*CCL4*), *CCL7*, *CCL13*, and their receptors: *CCR3*, *CCR4*, and *CCR6*; and common mutation genes including: *KRAS proto-oncogene*, *GTPase* (*KRAS*), *SMAD family member 4* (*SMAD4*) (**Figures 7A–E**). The positive interaction between the *FAM83A* axis expression, the immune landscape, and gene mutations will assist future immunotherapy for patients with PC.

## Discussion

Malignant pancreatic tumors are increasingly threatening human life and health and imposing a serious disease burden on society worldwide (1, 5). The very high mortality rate in PC places importance on exploring novel clinical biomarkers and staging systems to provide personalized treatments to improve patients’ long-term survival (1). Several studies showed that *FAM83A* plays a pivotal role in the tumorigenesis of various malignancies (12, 14, 21), however, its role in PC is poorly understood. In this study, we revealed FAM83A is associated with poor long-term survival and immune cell infiltration in pancreatic cancer. FAM83A may be used as a novel survival-related and immune-related biomarker. It suggests that FAM83A may be a novel therapeutic target for combined or individual treatment for patients with pancreatic cancer.

Many recent studies have demonstrated that ncRNAs (lncRNA, miRNA, and circular RNA) involved in tumorigenesis affect the expression level of target genes *via* the ceRNA action mechanism (31–33). Eleven candidate upstream miRNA binding targets of *FAM83A* were determined using seven prediction programs in the Starbase database. Correlation analysis between the candidate miRNAs and *FAM83A* identified *hsa-miR-129-5p* as the most adaptive upstream miRNA of *FAM83A* which suppresses its expression. Previous studies showed that *hsa−miR−129−5p* plays a vital regulatory role in glioma (34) and is involved in chemoresistance in ovarian cancer cells (35). However, few studies have focused on the potential mechanism of *hsa−miR−129−5p* in regulating target genes and tumorigenesis of PC. Our findings are the first to show that *hsa−miR−129−5p* is a candidate miRNA that negatively regulates *FAM83A* expression and participates in PC suppression.

ceRNA hypothesis states that lncRNAs are positively correlated with mRNA expression since they negatively regulate miRNA expression. Thus, lncRNAs of hsa−miR−129−5p/FAM83A axis is potentially positively correlated with carcinogenesis in PC. One hundred and eighteen potential lncRNAs of hsa−miR−129−5p/FAM83A axis was predicted, with only *AL049555.1* detected as the candidate lncRNA following correlation, survival, and differential expression analysis. *AL049555.1* is a novel lncRNA so its mechanism of action in human cancers has not been studied. This is the first study showing that AL049555.1/hsa−miR−129−5p/FAM83A axis is a vital ceRNA regulatory network involved in promoting PC development.

Various studies demonstrated that immune cells play a pivotal role in the tumor immune microenvironment which may affect the response to immunotherapy (36, 37) and the rate of ICI directly affects patient prognosis (38, 39). This study showed that *FAM83A* variation significantly influenced the ICI landscape of PC through significantly positive correlations with memory B cells and M0 macrophage infiltrations, with significant changes in B cells, CD4+ T cells, and CD8+ T cell infiltration levels.

Several immune-related genes including the immune checkpoint are closely related to the development of malignant tumors and affect the response to immunotherapy in patients with PC. For instance, targeted inhibition of programmed cell death 1 (PD-1) or programmed cell death ligand 1 (PD-L1) successfully treated various tumors (40, 41). This study showed that *FAM83A* expression correlated with various immune-related biomarkers such as *PDCD1* which suggests that targeting *FAM83A* may influence the response to immunotherapy in PC.

High mutation rates of *KRAS* and tumor protein p53 (*TP53*) are highly related to the immune status of tumors suggesting that they are predictors of responses to PD1/PDL1 immunotherapy (42, 43). This study showed that upregulated *FAM83A* positively correlated with *KRAS* and *mucin 16*, *cell surface associated* (*MUC16*), and negatively correlated with *SMAD4* and *titin* (*TTN*) indicating that targeting *FAM83A* may affect the key gene mutation proteins and further influence immunotherapy responses in PC.

In present study, four endpoints (OS, DSS, DFI, and PFI) survival analyses of several cancer types indicated that PC patients with high FAM83A expression had shorter long-term survival. The clinical prognostic values of *FAM83A* in PC carried out using a correlation analysis between *FAM83A* expression and clinicopathological characteristics showed increased *FAM83A* expression at later AJCC stages in PC. A nomogram was constructed and validated, providing novel predictive tools for clinicians showing that *FAM83A* expression and the N stage are independent risk factors using univariate and multivariate Cox regression.

Despite these significant findings, some limitations must be noted. First, all the obtained transcriptome data were analyzed retrospectively based on a multi database with intrinsic bias. Although, *in vitro* experiment validated *FAM83A* expression in tumorigenic PC tissues and in silico analysis propose a novel AL049555.1/hsa−miR−129−5p/FAM83A axis in tumorigenesis and reveal the axis-related immune microenvironment status in patients with PC, our results will need to be validated in a large independent cohort. Therefore, further abundant basic laboratory experiments and clinical trials are required to validate this work.

## Conclusion

**Figure 8:**
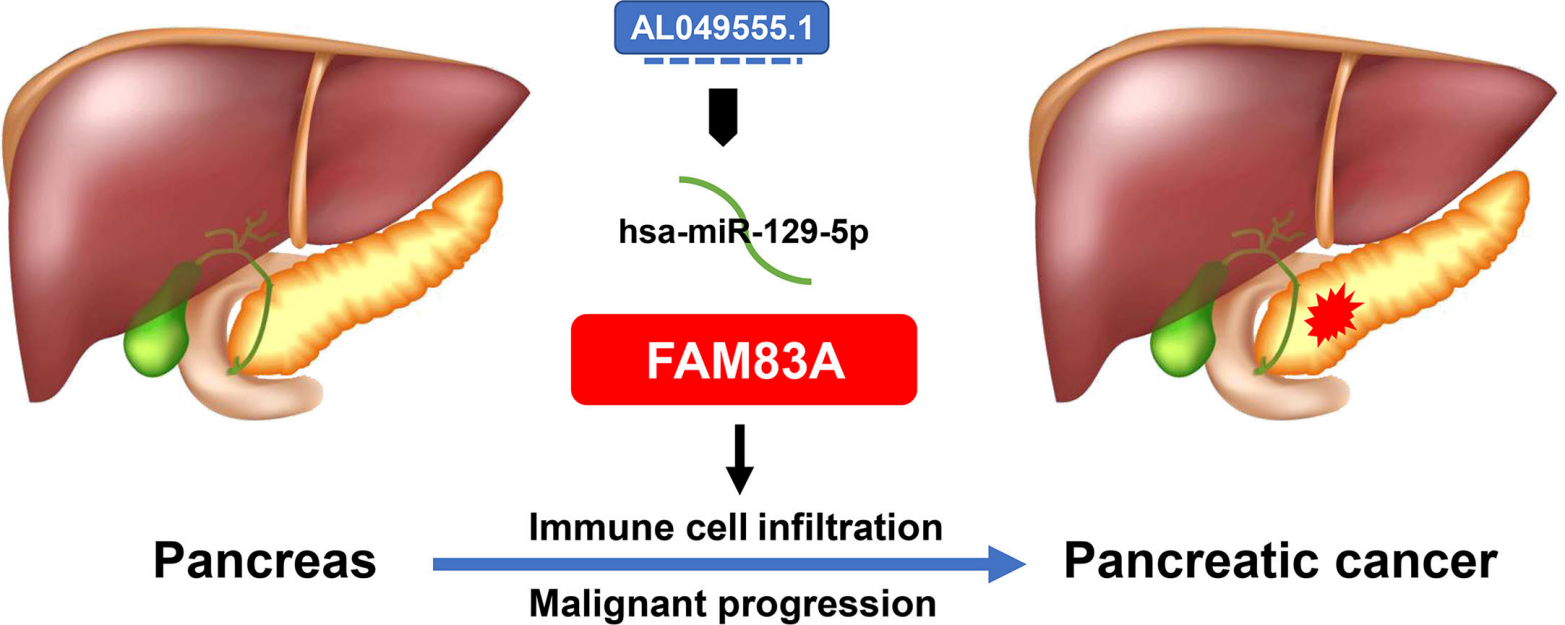
The model of AL049555.1/hsa−miR−129−5p/FAM83A axis in tumorigenesis of PC.

A novel PC AL049555.1/hsa−miR−129−5p/FAM83A axis was constructed (**Figure 8**). FAM83A accelerated tumorigenesis and development and executed its carcinogenic role by influencing ICI, immune-related biomarker expression, and expression of commonly mutated genes.

## Data availability statement

The raw data supporting the conclusions of this article will be made available by the authors, without undue reservation.

## Ethics statement

The studies involving human participants were reviewed and approved by the ethics committee of the No.924 Hospital of PLA Joint Logistic Support Force. The patients/participants provided their written informed consent to participate in this study.

## Author contributions

Study concept and design: GH, WZ. Drafting of the manuscript: GH, WZ, HW. Acquisition of data, analysis, and interpretation of data: HW, DW, YW, KZ, YL, GC. Critical revision of the manuscript: GH. Statistical analysis: HW, YF, JL. Study supervision: GH. All authors contributed to the article and approved the submitted version.
