# Supplementary material for: ncRNA-mediated upregulation of FAM83A is associated with poor prognosis and immune infiltration in pancreatic cancer
Source: Front Endocrinol (Lausanne). 2023 Mar 31;14:1093042. doi: 10.3389/fendo.2023.1093042 (PMC10102663; doi:10.3389/fendo.2023.1093042)
Supplement: Supplementary file 1 [file Image_1.pdf]

Supplementary materials

ncRNA-mediated upregulation of FAM83A is associated with poor prognosis and immune infiltration in pancreatic cancer

Supplementary FigureS1

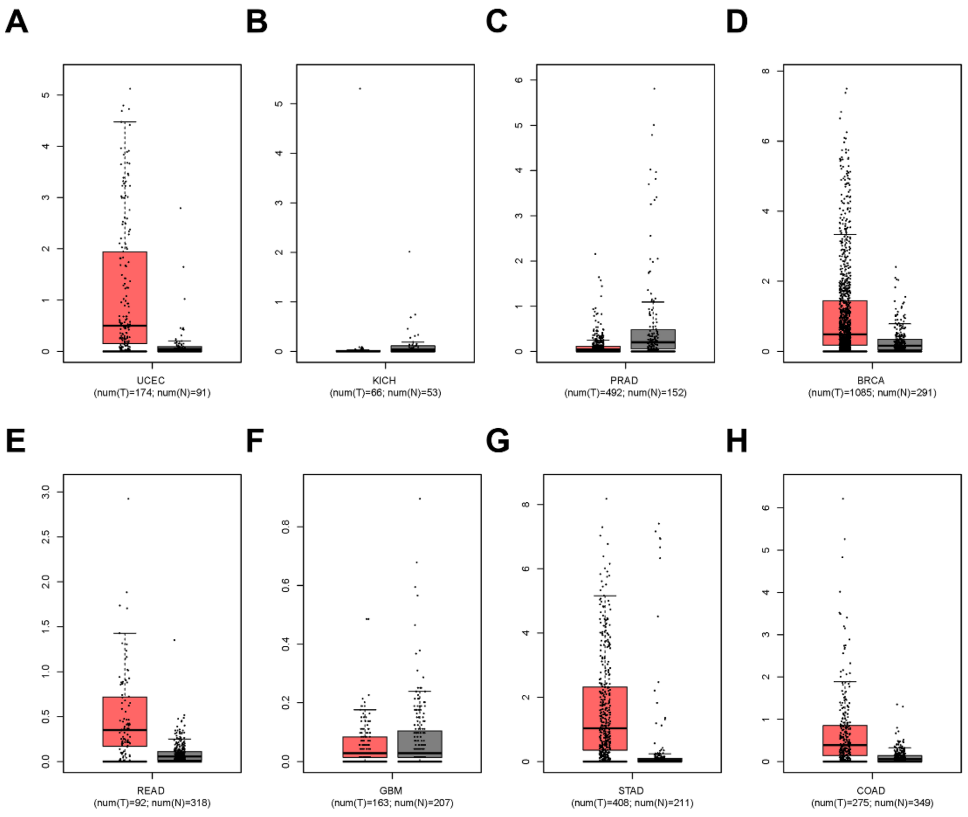

sFigure 1: Expression analysis for FAM83A in multiple cancers
(A-H) The differential expression of FAM83A between tumor and normal tissues in TCGA and GTEx. (A) UCEC (B), KICH (C), PRAD (D), BRCA (E), READ (F), GBM (G), STAD. (H), COAD *p value < 0.05; **p value < 0.01; ***p value < 0.001.

Supplementary FigureS2

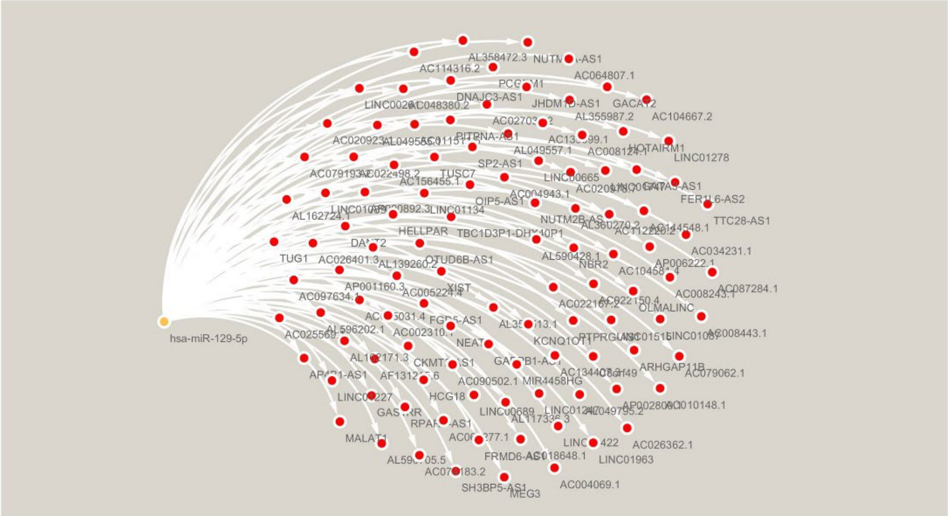

sFigure 2: Identification of potential upstream miRNAs of hsa-miR-129-5p in PC based on starbase database.

Supplementary FigureS3

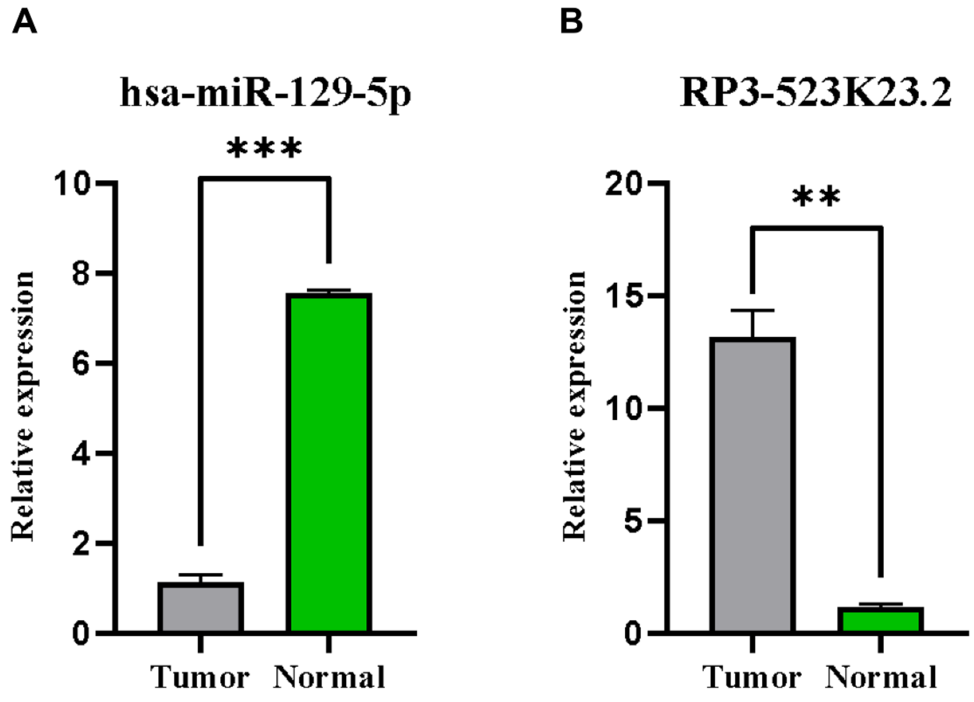

sFigure 3: (A) The qRT-PCR result of hsa-miR-129-5p between the tumor and normal tissues. (B) The qRT-PCR result of AL049555.1 between the tumor and normal tissues.
